# Reproducibility of adenosine stress cardiovascular magnetic resonance in multi-vessel symptomatic coronary artery disease

**DOI:** 10.1186/1532-429X-12-42

**Published:** 2010-07-21

**Authors:** Sharon Chih, Peter S Macdonald, Michael P Feneley, Matthew Law, Robert M Graham, Jane A McCrohon

**Affiliations:** 1 Victor Chang Cardiac Research Institute, Lower Packer Building, Liverpool Street, Sydney, Australia; 2 Department of Cardiology, St Vincent's Hospital, Victoria Street, Sydney, Australia; 3 University of New South Wales, Sydney, Australia

## Abstract

**Purpose:**

First-pass perfusion cardiovascular magnetic resonance (CMR) is increasingly being utilized in both clinical practice and research. However, the reproducibility of this technique remains incompletely evaluated, particularly in patients with severe coronary artery disease (CAD). The purpose of this study was to determine the inter-study reproducibility of adenosine stress CMR in patients with symptomatic multi-vessel CAD and those at low risk for CAD.

**Methods:**

Twenty patients (10 with CAD, 10 low risk CAD) underwent two CMR scans 8 ± 2 days apart. Basal, mid and apical left ventricular short axis slices were acquired using gadolinium 0.05 mmol/kg at peak stress (adenosine, 140 μ/kg/min, 4 min) and rest. Myocardial perfusion was evaluated qualitatively by assessing the number of ischemic segments, and semi-quantitatively by determining the myocardial perfusion reserve index (MPRi) using a normalized upslope method. Inter-study and observer reproducibility were assessed--the latter being defined by the coefficient of variation (CoV), which was calculated from the standard deviation of the differences of the measurements, divided by the mean. Additionally, the percentage of myocardial segments with perfect agreement and inter- and intra-observer MPRi correlation between studies, were also determined.

**Results:**

The CoV for the number of ischemic segments was 31% with a mean difference of -0.15 ± 0.88 segments and 91% perfect agreement between studies. MPRi was lower in patients with CAD (1.13 ± 0.21) compared to those with low risk CAD (1.59 ± 0.58), p = 0.02. The reproducibility of MPRi was 19% with no significant difference between patients with CAD and those with low risk CAD (p = 0.850). Observer reproducibility for MPRi was high: inter-observer CoV 9%, r = 0.93 and intra-observer CoV 5%, r = 0.94. For trials using perfusion CMR as an endpoint, an estimated sample size of 12 subjects would be required to detect a two-segment change in the number of ischemic segments (power 0.9, α 0.05).

**Conclusions:**

Adenosine stress CMR, by qualitative and semi-quantitative normalized upslope analyses are reproducible techniques in both patients with multi-vessel CAD and those without known CAD. The robust inter-study reproducibility of perfusion CMR supports its clinical and research application.

## Background

First-pass perfusion cardiovascular magnetic resonance (CMR) allows non-invasive, rapid and ionizing-radiation-free evaluation of myocardial perfusion and, in combination with other CMR techniques, provides a comprehensive assessment of ischemic heart disease. Stress CMR has high diagnostic accuracy with 91% sensitivity and 81% specificity reported for the detection of coronary artery disease (CAD) [1]. Whilst single photon emission computed tomography (SPECT) has been the most widely utilized myocardial perfusion imaging modality, CMR has demonstrated superiority for the detection of CAD [2-4]. In particular, the improved spatial resolution of CMR permits delineation of both small and diffuse areas of subendocardial ischemia in the setting of mild to moderate CAD and multi-vessel disease. In the largest multicenter clinical trial to date, the performance of CMR was shown to be comparable to SPECT, and possibly superior in the detection of three-vessel CAD [4]. Furthermore, there is also emerging evidence for CMR as a powerful coronary risk stratification tool. In patients with known or suspected CAD, a normal stress CMR study was shown to predict a 0.8% risk of cardiac death or non-fatal myocardial infarction (MI) over a three year period, whilst an abnormal result was shown to have a risk proportional to the degree of abnormality [5]. In another study of patients presenting to the emergency department with chest pain and a negative troponin, an abnormal perfusion CMR scan had a 100% sensitivity and 91% specificity for predicting CAD or an adverse cardiac event at one year [6].

However, despite the appeal and anticipated future application of perfusion CMR in both clinical and research arenas, its reproducibility remains incompletely evaluated, particularly in the severe CAD patient-population. Reproducibility is a measure of the ability of a test to produce the same result when applied under similar conditions. Thus, reproducibility affects test precision, reliability and is particularly important for tests that are used in serial examinations to evaluate response to therapy or for clinical monitoring. Knowing the inherent variability of a technique is critical for interpreting the significance of changes in measurements. For research applications, tests with poor reproducibility require increased sample sizes to reduce statistical error, which would unfavorably increase study duration and costs.

Thus far, only three small studies have reported the reproducibility of perfusion CMR [7-9]. In a study of 16 subjects (7 volunteers and 9 CAD patients), 21% and 41% coefficient of variation (CoV) were reported for adenosine stress CMR myocardial perfusion reserve index (MPRi) by Fermi deconvolution and normalized upslope analysis, respectively [7]. Muhling et al. [8] reported good intra- (*R *= 0.80-0.85) and inter-observer (*R *= 0.83-0.88) agreement for good quality images, using the quantitative Fermi deconvolution method. In another study of the qualitative analysis of dobutamine stress CMR in patients with severe Canadian Cardiovascular Society class III-IV angina, an inter-observer agreement, K, of 0.70, and low intra-subject variability were reported [9]. These small studies combined with high CMR diagnostic performance suggest that perfusion CMR has reasonable reproducibility. Further confirmation is, however, prudent, particularly in clinical research where perfusion CMR is increasingly being employed as a clinical endpoint and accurate estimates of sample size are necessary. Furthermore, in addition to technical factors, reproducibility may also be affected by biological conditions related to the subject, and observer variability associated with subjective assessments. Thus, reproducibility needs to be evaluated in specific patient cohorts and also considered in individual imaging centres due to varying local reporter-expertise, imaging and contrast administration protocols. The purpose of this study was to (1) determine the reproducibility of qualitative and semi-quantitative analyses of first-pass perfusion adenosine stress CMR in patients with multi-vessel CAD versus those at low risk of CAD and; (2) determine sample sizes required for trials utilizing CMR myocardial perfusion as the primary end-point.

## Methods

The study was conducted between January and September 2009 with the approval of the St Vincent's Hospital Human Research Ethics Committee and with written informed consent from all patients.

### Study Design

Patients underwent two CMR scans that were performed on two separate occasions up to two weeks apart. CMR scans were analyzed in random order by two reporters (JM and SC) who were blinded to patients' details. Qualitative analysis was performed by consensus reporting and semi-quantitative analysis was performed by a single reporter (SC). Inter-study reproducibility was assessed by comparing each patient's first and second scans. Additionally, a single short-axis slice (SA) was chosen from 20 randomly selected scans (10 case and 10 control) and semi-quantitative analysis repeated by the same observer (SC) and by a second observer (JM) to assess intra- and inter-observer reproducibility, respectively.

### Patient Population

Subjects were recruited into one of two groups: CAD (case) or low risk CAD (control). Patients with CAD had angiographically documented multi-vessel CAD (≥70% stenosis in ≥2 major coronary vessels (>2 mm diameter)) and Canadian Cardiovascular Society class II-IV angina. Classification of patients in the low risk CAD group was based on them having a Framingham estimated 10 year coronary heart disease risk of <10% [10]. Exclusion criteria were alteration in CAD disease management or a new coronary event in the period between the two CMR examinations, contraindication to CMR including incompatible implants or severe claustrophobia, contraindication to adenosine including severe aortic stenosis, conduction disorder (2:1 or greater atrioventricular block) or severe bronchospasm, atrial fibrillation, uncontrolled heart failure, unstable angina or myocardial infarction within 7 days, and significant renal impairment (GFR ≤ 60 mL/min).

### Cardiovascular Magnetic Resonance Imaging

CMR was performed at St George Hospital (Kogarah) and Specialist Magnetic Resonance Imaging (Camperdown), New South Wales on a 1.5 Tesla scanner (Philips Intera, Best, The Netherlands) using a five channel cardiac phased-array coil, cardiac gating and repeated breath holds. Each examination involved an assessment of resting ventricular function, first-pass perfusion imaging and late gadolinium enhancement (LGE) imaging for infarct delineation.

Cardiac functional imaging was assessed by steady-state free precession cine imaging (balanced turbo field echo) in the long and short axis (SA) planes. The basal SA slice was positioned at the level of the atrioventricular valve in end-diastole, with all subsequent cines acquired in 8 mm steps towards the apex. Parameters were: echo time = 1.82 ms, repeat time = 3.6 ms, field of view = 350 × 280 mm, read matrix = 192, phase matrix = 131, frames = 18 heart phases, flip angle = 70°.

**Figure 1:**
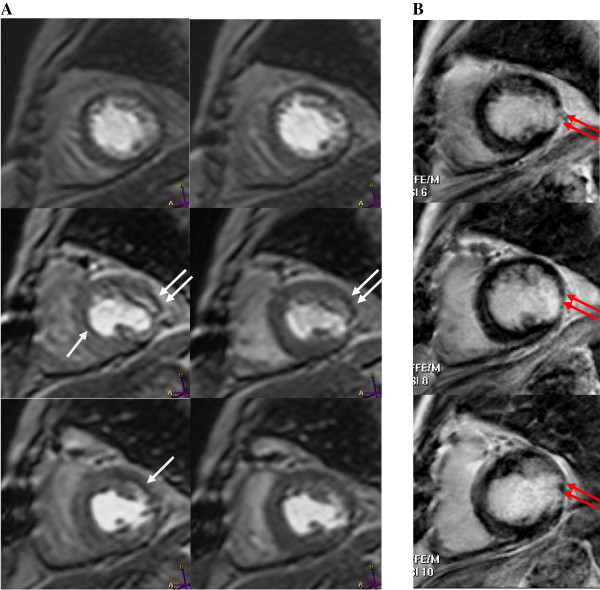
**Myocardial first pass perfusion imaging**. **A**, Apical (top row), mid (middle row) and basal (bottom row) short axis slices acquired at stress (left) and rest (right). Inducible ischemia demonstrated by myocardial hypoenhancement, which is visible at stress (single arrow) and absent at rest. Fixed perfusion defects present at stress and rest (white double arrows) corresponding to areas of infarction on **B**, Late gadolinium enhancement images. Basal to mid lateral infarction (red double arrows).

Myocardial perfusion assessment was performed using a T1-weighted single shot gradient echo sequence with a saturation recovery pre-pulse. Parameters were: echo time = 1.06 ms, repetition time = 3 ms, field of view = 360 × 360 mm, read matrix = 128, phase matrix = 97, reconstruction matrix = 240, voxel size = 2.8 mm × 3.6 mm × 8 mm, flip angle = 20°. Images were acquired over 50 dynamic scans in standard basal, mid and apical SA slices (Figure 1A). LGE imaging for infarct evaluation was performed using an inversion recovery gradient-echo sequence with inversion time optimized to null normal myocardium (Figure 1B).

First-pass perfusion was assessed using three 0.05 mmol/kg boluses of gadolinium (Magnevist, Bayer Schering Pharma), administered at peak stress, 10 minutes later at rest (Figure 1A) and immediately after completion of rest imaging (Figure 1B). Gadolinium was delivered via a power injector (Medrad Spectris) at 5 ml/sec with a 15 ml saline flush. Stress was induced with an intravenous infusion of 140 μg/kg/min adenosine (Adenoscan, Sanofi-Synthelabo), commenced up to four minutes prior to stress image acquisition. To ensure maximal vasodilatory responses to adenosine, patients were asked to abstain from caffeinated beverages for 24 hours prior.

#### Image Analyses

**Figure 2:**
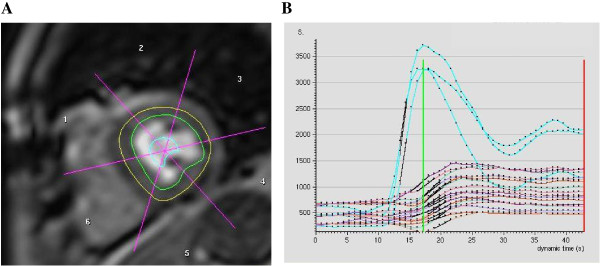
**Semi-quantitative perfusion analysis**. Semi-quantitative perfusion analysis: **A**, Manual tracing of the endocardial (green) and epicardial (yellow) borders and division of the LV myocardium into 6 segments, and **B**, Generation of signal intensity (y axis) vs. time (x axis) curves for each myocardial segment and LV (blue). Maximal upslopes for each myocardial segment, normalized to LV input, with the myocardial perfusion reserve index (MPRi) as the ratio of stress to rest upslopes.

Image analysis was performed offline on a Philips View Forum workstation using cardiac analysis software. Segmental analysis was based on an 18-segment model: radial division of each of the three SA slices into six segments with positioning of segment 6 at the insertion of the right ventricle into the inferior septum of the LV (Figure 2A). Image quality was graded as 1 = suboptimal, 2 = adequate, 3 = good. Measurements for each parameter were reported as a global score (all three SA slice) and according to left anterior descending (LAD), circumflex (Cx) and right coronary artery (RCA) territories as per AHA guidelines.

LV volumes, function and mass were derived by manual tracing of epicardial and endocardial borders of contiguous SA slices at end-diastole and end-systole. The end-systolic phase was visually determined as the one with the smallest cavity area and this was then used for all LV levels. Papillary muscles and trabeculae with visible borders in continuity with the endocardium were included in both mass and volume analyses. Perfusion was assessed qualitatively and semi-quantitatively. An automated image stabilizer was applied to correct for gross cardiac motion and images were windowed appropriately to optimize visualization of perfusion defects, with the same parameters used for both stress and rest images. Qualitative analyses were performed by visual assessment of myocardial signal enhancement in each segment. A perfusion defect was defined as any visible hypoenhancement appearing after maximum signal intensity in the LV blood pool (three frames after the arrival of contrast in the LV cavity) and persisting for at least four dynamics. Perfusion defects were graded by their transmural extent (1 = normal, 2 = subendocardial defect, 3 = transmural defect) and degree of hypoenhancement (1 = normal (bright), 2 = probably abnormal (grey defect), 3 = definitely abnormal (black defect)). Segments affected by artefact were graded as 0. An artefact was defined as a defect present at rest but absent at stress, matching stress and rest defects without evidence of infarct on LGE, or segments in the basal SA slice obtained through the left ventricular outflow tract. Transmural and hypoenhancement ischemia indices, reflecting the degree of reversible ischemia, were calculated as a ratio of summed segmental stress-to-rest scores, thus resulting in a grading scale of 1 = no ischemia, >1 and <3 = mild ischemia, and 3 = severe ischemia. Abnormal ischemic segments were defined by segmental transmural ischemia index >1. Myocardial perfusion ratio index (MPRi) was derived from maximal upslopes of stress to rest myocardial contrast signal intensity time curves, normalized to LV input (Figure 2B). Myocardial segments in the basal slice obtained through the LV outflow tract were excluded. Segmental infarction was graded as 1 = normal, 2 = subendocardial infarct and 3 = transmural infarct. The infarct index was determined by the summed segmental infarct score divided by the number of segments analyzed.

### Statistical Analyses

Within group differences in adenosine dose, hemodynamic parameters and baseline CMR measures were assessed using paired Student's t-test, and differences in symptomatic response using Fisher's exact test. Reproducibility was defined by the coefficient of variation (CoV) [11]. This was determined by dividing the standard deviation (SD) of the differences between the two measurements by the average of the two measurements, and was expressed as a percentage. For a continuous variable, inter-study reproducibility between groups was assessed by comparing the squared difference of the two measurements between case and control subjects, with statistical significance being assessed by the Mann-Whitney U test. Where the squared difference of the two measurements equaled zero, half of the next smallest value was entered. For perfusion ordinal data, reproducibility was also assessed by calculating the percentage of reported segments with perfect agreement in ischemia grading (none, mild or severe) between the two studies. Bivariate correlations were calculated using the Spearman correlation coefficient to assess inter-study, inter- and intra-observer agreement for semi-quantitative analyses. Sample sizes were calculated to detect of varying degrees of change in perfusion parameters at an α level of 0.05 and power of 0.9 (http://hedwig.mgh.harvard.edu/sample_size/size.html). All data were analyzed on SPSS for Windows, release 17.0 (SPSS Inc., Chicago, Illinois).

## Results

### Patient Characteristics

Twenty patients (10 low risk CAD controls and 10 patients with CAD) were recruited. Patient characteristics are shown in Table 1. Patients in the control group were younger, without prior cardiac history and had a mean Framingham risk score of 7 ± 3%. Patients with CAD had an average of 2.8 ± 0.4 native coronary arteries diseased, were symptomatic with CCS class II or III angina and the majority (n = 9, 90%) had suffered a previous myocardial infarct.

**Table 1:** Patient demographics.

Characteristic	Case (n = 10)	Control (n = 10)
Age, years	68 ± 13 (39 - 83)	56 ± 4 (52 - 62)*
Male	8 (80)	8 (80)
**Vascular Risk Factors**		
Body mass index, kg/m^2^	26.6 ± 6.8 (24.0 - 33.0)	25.9 ± 3.5 (20.4 - 31.5)
Diabetes	4 (40)	0 (0)
Hypertension	4 (40)	3 (30)
Dyslipidemia^†^	8 (80)	1 (10)
Smoking previous/current	6 (60)/1 (10)	4 (40)/0 (0)
Framingham risk score, %	-	7 ± 3 (2 - 10)
**Cardiac History and Status**		
CCS class angina class II/III	4 (40)/6 (60)	-
Myocardial infarction	9 (90)	-
Number of diseased native vessels	2.8 ± 0.4 (2 - 3)	-
Number of CABG operations	0.7 ± 0.5 (0 - 1)	-
Number of PCI	1.3 ± 1.5 (0 - 5)	-
Number of cardiac medications daily	6.9 ± 1.8 (4 - 8)	-

Values are number (%) or mean ± SD (range). CABG = coronary artery bypass graft; CCS = Canadian Cardiovascular Society; PCI = percutaneous coronary intervention

*p < 0.05 for control vs. case.

^†^Presence of hyperlipidemia or lipid-lowering therapy.

### Adenosine tolerance and hemodynamic effects

Adenosine-related side effects were reported by the majority of patients (Table 2). The adenosine infusion was discontinued if the patient developed severe angina, arrhythmias, hypotension or at the patient's request. Adenosine dosage did not differ significantly between studies in either the case or control group, but was administered for longer in the control compared to case group: 2.20 ± 0.63 vs. 1.65 ± 0.55 minutes, p = 0.050.

**Table 2:** Adenosine tolerance and hemodynamic effects.

	Control			Case		
	
Parameter	Study 1	Study 2	p	Study 1	Study 2	p
**Rest**						
Heart rate, beats/min	60 ± 7	67 ± 10	0.411	59 ± 9	61 ± 12	0.317
Systolic BP, mmHg	130 ± 13	130 ± 11	0.912	138 ± 25	139 ± 25	0.874
Diastolic BP, mmHg	81 ± 16	82 ± 12	0.787	82 ± 11	70 ± 9	0.004
**Peak stress**						
Heart rate, beats/min	82 ± 16	83 ± 10	0.883	76 ± 19	70 ± 12	0.063
Systolic BP, mmHg	131 ± 12	129 ± 12	0.537	130 ± 31	135 ± 20	0.671
Diastolic BP, mmHg	82 ± 16	83 ± 10	0.134	71 ± 14	71 ± 15	1.000
**Adenosine infusion**						
Duration, min	2.3 ± 0.6	2.2 ± 0.7	0.583	1.7 ± 0.5	1.7 ± 0.6	0.204
Chest pain	4 (40)	4 (40)	0.133	5 (50)	4 (40)	0.524
Breathlessness	4 (40)	1 (10)	0.400	5 (50)	5 (50)	0.206
Flushing, headache, dizziness	8 (80)	8 (80)	0.467	3 (30)	3 (30)	0.183

Values are number (%) or mean ± SD. BP = blood pressure.

### Cardiac function and perfusion

There were no significant differences between control and case patients for baseline LV function, volumes or mass (Table 3). As expected, myocardial perfusion was reduced across all measures in the CAD compared to the control group (p < 0.020 for all measures). In the CAD group, infarction as evidenced by LGE, was detected in 29% (45 of 156 segments) of all myocardial segments analyzed. In this group, mean global infarct index was 1.35 ± 0.34 and was comparable across all three coronary artery territories: LAD = 1.22 ± 0.28, Cx = 1.45 ± 0.49, RCA = 1.42 ± 0.54.

**Table 3:** Baseline left ventricular cardiac function and perfusion.

Parameter	Control	Case
**Left ventricular volumes and function**		
Ejection Fraction, %	69 ± 8	65 ± 10
End diastolic volume, ml	146 ± 17	139 ± 40
End systolic volume, ml	47 ± 15	50 ± 27
Stroke volume, ml	99 ± 11	88 ± 21
Mass, g	113 ± 22	126 ± 23
**Myocardial Perfusion - Global**		
Myocardial perfusion reserve index*	1.59 ± 0.58^#^	1.13 ± 0.21
Number of ischemic segments^†^	0.75 ± 1.53^#^	4.60 ± 2.46
Transmural ischemia index^‡^	1.10 ± 0.26^#^	1.33 ± 0.19
Hypoenhancement ischemia index^§^	1.18 ± 0.42^#^	1.51 ± 0.23
Infarct index^||^	1.00 ± 0.00^#^	1.35 ± 0.34

Values are mean ± SD. Measurements are an average of the first and second study.

*Averaged segmental stress:rest maximal normalized upslopes.

^†^Number of myocardial segments with reversible ischemia by qualitative analysis.

^‡^Summed segmental transmural stress:rest score (1 = normal, 2 = subendocardial defect, 3 = transmural defect).

^§^Summed segmental hypoenhancement stress:rest score (1 = normal, 2 = grey defect, 3 = black defect).

^||^Summed segmental infarct score divided by number of segments analyzed (1 = normal, 2 = subendocardial infarct, 3 = transmural infarct).

^#^p < 0.020 for control vs. case.

### Inter-study reproducibility

The two CMR studies were performed at a mean of 7.7 ± 2.2 days apart. A total of 40 scans were performed, providing 104 slices (624 segments) for analysis with equal numbers from case and control groups. In four patients, only two slices were analyzable due to a high heart rate preventing acquisition of all three slices (n = 2), breath-holding problems resulting in the apical slice being positioned too distally (n = 1) and LV outflow tract affecting the basal slice (n = 1). In two patients, only a single slice was analyzed due to a combination of high heart rate and LV outflow tract acquisition in one patient, and gating difficulties in another patient. Overall, image quality for perfusion analysis was considered adequate in all patients with a mean grading of 2.7 ± 0.5 for the first study and 2.8 ± 0.4 for the second study. No significant differences in image quality were detected between case and control groups or between the two studies within each group.

#### LV volume and function

As shown in Table 4, inter-study reproducibility was consistently high for LV volumes, ejection fraction and mass in both case and control groups. A comparison between control and CAD groups revealed a statistically significant difference in reproducibility only for LV end-diastolic volume, which was slightly lower for CAD compared to control patients: 4.4% vs. 1.9% CoV for case and control group, respectively (p = 0.018).

**Table 4:** Inter-study reproducibility for LV volumes and function.

	Coefficient of Variation (%)		
		
Parameter	Control	Case	p*
Left ventricular ejection fraction	2.4	2.7	0.292
Left ventricular end diastolic volume	1.9	4.4	0.018
Left ventricular end systolic volume	6.8	6.4	0.787
Left ventricular stroke volume	2.1	4.2	0.483
Left ventricular mass	4.4	4.0	0.619

*Comparison of CoV between control and case.

#### Qualitative myocardial perfusion analysis

Reproducibility for all qualitative measures of myocardial perfusion was high, as demonstrated by consistently low CoVs, high percentage of perfect agreement between studies, and small mean difference between studies (Table 5). Reproducibility of perfusion defect assessment by evaluation of transmural extent (CoV 8.1%) and degree of hypoenhancement (CoV 9.4%) were both high; however, the percentage of perfect agreement between studies was higher in the former. Mean difference in number of ischemic myocardial segment between studies was small: -0.10 ± 0.57 for control and -0.20 ± 1.14 for CAD. Reproducibility varied across different coronary artery territories and was generally lower compared to global analysis, and in the CAD compared to control group.

**Table 5:** Inter-study reproducibility for qualitative perfusion parameters.

	All Subjects			Control			Case		
	
Parameter	Mean Difference*	CoV	Perfect Agreement^†^	Mean Difference*	CoV	Perfect Agreement^†^	Mean Difference*	CoV	Perfect Agreement^†^
**Number of ischemic segments**									
Global	-0.15 ± 0.88	30.6	90.7	-0.10 ± 0.57	*75.7*^‡^	96.2	-0.20 ± 1.14	24.7	85.3
LAD	-0.00 ± 0.65	72.1	89.3	-0.10 ± 0.74	*210.8*^‡^	95.2	-0.10 ± 0.57	39.2	83.3
Circumflex	-0.05 ± 0.60	78.0	90.3	-0.10 ± 0.32	*210.8*^‡^	96.2	0.00 ± 0.82	58.3	84.3
RCA	-0.07 ± 0.70	41.0	93.0	-0.10 ± 0.32	*126.5*^‡^	97.6	-0.25 ± 0.89	27.6	88.6
**Transmural ischemia index**									
Global	-0.03 ± 0.10	8.1	90.7	-0.04 ± 0.11	9.7	96.2	-0.03 ± 0.09	7.1	85.3
LAD	-0.05 ± 0.29	22.8	89.3	0.00 ± 0.15	13.3	95.2	0.11 ± 0.38	26.7	83.3
Circumflex	-0.04 ± 0.21	17.6	90.3	-0.08 ± 0.18	16.5	96.2	0.01 ± 0.23	18.1	84.3
RCA	-0.04 ± 0.17	13.4	93.0	-0.02 ± 0.06	5.6	97.6	-0.07 ± 0.24	16.8	88.6
**Hypoenhancement ischemia index**									
Global	-0.04 ± 0.13	9.4	81.1	-0.01 ± 0.06	5.4	94.2	-0.07 ± 0.17	11.1	67.9
LAD	0.06 ± 0.39	28.5	78.9	0.06 ± 0.14	12.1	90.3	-0.04 ± 0.25	35.1	67.2
Circumflex	-0.05 ± 0.22	17.3	86.4	-0.07 ± 0.21	18.6	98.1	-0.08 ± 0.24	17.0	74.5
RCA	-0.11 ± 0.25	16.8	77.9	-0.07 ± 0.14	11.0	95.2	-0.06 ± 0.28	19.2	61.4
**Infarct index**									
Global	-0.00 ± 0.06	4.7	96.0	0.00 ± 0.00	0.0	100.0	0.00 ± 0.08	6.1	94.2
LAD	0.06 ± 0.39	4.2	95.4	0.00 ± 0.00	0.0	100.0	0.00 ± 0.07	5.5	96.7
Circumflex	-0.05 ± 0.22	6.4	97.1	0.00 ± 0.00	0.0	100.0	-0.03 ± 0.11	7.7	94.2
RCA	-0.11 ± 0.25	9.6	95.3	0.00 ± 0.00	0.0	100.0	0.04 ± 0.17	11.8	90.9

Values are mean ± SD, CoV (%), perfect agreement (%). LAD = left anterior descending; RCA = right coronary artery.

*Mean difference between first and second study.

^†^Percentage of segments with perfect agreement in ischemia grading between the two studies.

^‡^CoV is a poor measure of reproducibility due to the average of measurements approximating zero.

#### Semi-quantitative myocardial perfusion analysis - MPRi

The inter-study reproducibility for MPRi is shown in Table 6. The between-study correlation for global MPRi was moderate: Spearman's correlation coefficient r = 0.76 (p < 0.001) for all subjects, r = 0.69 (p = 0.026) for control group and r = 0.68 (p = 0.029) for case group. MPRi CoV was higher compared to qualitative perfusion parameters. Regional coronary artery analysis was less reproducible compared to global analysis in the CAD group (global CoV 23% vs. regional 19%, p = 0.051) but comparable in the control group (global CoV 18% vs regional CoV 19%, p = 0.528). There was no difference in MPRi reproducibility between case and control groups (p = 0.821).

**Table 6:** Inter-study reproducibility for myocardial perfusion reserve index.

	All Subjects		Control		Case		
		
Parameter	Mean Difference*	CoV	Mean Difference*	CoV	Mean Difference*	CoV	p^†^
Global	0.07 ± 0.26	18.9	-0.01 ± 0.28	17.6	0.15 ± 0.22	18.8	0.850
LAD	0.07 ± 0.25	18.4	0.03 ± 0.28	18.1	0.12 ± 0.22	18.9	0.384
Circumflex	0.11 ± 0.21	19.2	0.02 ± 0.28	17.6	0.20 ± 0.22	19.4	0.623
RCA	0.01 ± 0.34	25.6	-0.10 ± 0.32	20.7	0.12 ± 0.33	30.6	0.821

Values are mean ± SD and CoV (%). CoV = coefficient of variation; LAD = left anterior descending artery; RCA = right coronary artery.

*Mean difference between first and second study.

^†^Comparison of CoV between control and case.

#### Inter-observer and intra-observer myocardial perfusion reproducibility

There was good agreement for MPRi between and within observers, with low CoVs and moderate to high correlation coefficients (Table 7). Reproducibility was lower for the CAD versus the control group for both, inter- and intra-observer analysis, with a trend towards statistical significance for inter-observer analysis (p = 0.056).

**Table 7:** Inter- and intra-observer reproducibility for MPRi.

	All Subjects		Control		Case		
		
	CoV	r	CoV	r	CoV	r	p*
Inter-observer	9.0	0.93^†^	5.0	0.99^†^	13.2	0.79^†^	0.056
Intra-observer	5.3	0.94^†^	4.2	0.96^†^	7.0	0.76^†^	0.733

CoV (%) = coefficient of variation. r = Spearman's correlation co-efficient.

*Comparison of CoV between control and case.

^†^p < 0.02

### Sample size estimation

Within-subject variability estimates for the number of ischemic segments, transmural ischemia index and MPRi are shown by the standard deviation of between-study differences for these parameters (Table 8). As shown, clinical trials utilizing CMR to detect changes in myocardial perfusion would require small sample sizes. For example, it is estimated that a two-group parallel-design trial would require 12 patients (6 vs. 6) to detect a mean difference of two abnormal segments or 74 patients (37 vs. 37) to detect a 0.20 change in MPRi with 90% power (2-alpha = 5%).

**Table 8:** Sample size estimates for myocardial perfusion by CMR.

Patient Group	SD*	Sample Size^†^
**All subjects**		
2 segment change in number of ischemic segments	0.88	12
0.10 change in transmural ischemia index	0.10	46
0.20 change in myocardial perfusion reserve index	0.26	74
**Low risk coronary artery disease - Control**		
2 segment change in number of ischemic segments	0.57	8
0.10 change in transmural ischemia index	0.11	54
0.20 change in myocardial perfusion reserve index	0.28	86
**Coronary artery disease - Case**		
2 segment change in number of ischemic segments	1.14	16
0.10 change in transmural ischemia index	0.09	38
0.20 change in myocardial perfusion reserve index	0.22	54

*Standard deviation of differences between first and second study.

^†^Sample size estimates for a two treatment parallel trial design.

## Discussion

This study demonstrates that adenosine stress perfusion CMR has good reproducibility for both qualitative and semi-quantitative analyses and in both patients with and without CAD. There is limited published data available for the reproducibility of serial myocardial perfusion imaging modalities, especially for CMR. The inter-study reproducibility of CMR has been demonstrated for LV volumes, ejection fraction and mass [11,12], but only three small studies have described the reproducibility of perfusion CMR. Of these, one study evaluated only observer reproducibility [8] and a second study used dobutamine as the pharmacological stress agent [9]. Elkington et al. [7] assessed the inter-study reproducibility for adenosine stress CMR in a cohort of 9 CAD patients and 7 healthy volunteers. They reported a CoV of 41% for transmural MPRi and 39% (inferior segment) to 55% (anterior segment) for regional analyses using the normalized upslope method. In contrast, we demonstrated improved reproducibility with an MPRi CoV of 19%, 18%, 19% and 26% for global, LAD-, Cx- and RCA-territory, respectively. Perfusion findings were generally less reproducible by regional coronary artery analysis compared to global analysis. This may be methodological as global and regional perfusion parameters are derived by summed segmental perfusion measurements divided by the number of segments analyzed (18 for global and 5 to 7 for regional coronary artery territory). Hence, segment variability in measurements between studies will be diluted to a greater extent for global versus regional analyses.

The reproducibility of qualitative analysis was better than that for semi-quantitative analysis by MPRi: global CoV for all subjects - 19% for MPRi, 8% for transmural ischemia index and 9% for hypoenhancement ischemia index. As fully automated techniques remove observer variability, improved accuracy and uniformity of measures are expected for quantitative compared to qualitative techniques. MPRi by normalized upslope analysis, however, is at best semi-quantitative as it is dependent on the operator to discern endocardial and epicardial borders accurately throughout the cardiac cycle. Furthermore, the occurrence of certain types of artefacts (eg. ectopy, stress and rest slice mismatch) may impede semi-quantitative analysis but may be overcome by an experienced reporter on visual inspection.

In comparison to control subjects, patients with known CAD were expected to have lower inter-study reproducibility for a number of reasons, including increased myocardial scarring from prior infarction, previous coronary interventions (PCI and CABG) generating artefacts, and also poorer clinical condition affecting testing due for example, to an increased susceptibility to ischemia induced arrhythmias or to an inability to breath-hold. As expected, LGE was not detected in the control group but was present in 29% of myocardial segments analyzed in the CAD group. The mean infarct index was comparable across all three coronary artery territories. Mean differences between studies were higher, and the proportion of myocardial segments with perfect agreement was lower for the CAD compared to the control group. However, despite the presence of moderate scar burden in the CAD compared with control group, there was no significant difference in MPRi reproducibility between the two groups (p = 0.850). This suggests that the presence of scar may affect qualitative but not semi-quantitative analysis. Intra-observer reproducibility was similar between the two groups (p = 0.733) and there was only a trend (p = 0.056) towards lower inter-observer reproducibility for MPRi in patients with CAD versus controls: CoV 5% vs. 13% for control and CAD, respectively. Elkington et al. [7] reported no significant differences in reproducibility between patients with CAD and normal subjects.

The small differences observed between the CAD and control groups in this study may be secondary to a lower than expected reproducibility in the control group. Our control group was selected on the basis of low estimated risk for CAD as defined by a Framingham risk score of <10%. Therefore, patients in this group may have had undiagnosed underlying CAD. Notably, the mean MPRi was 1.59 ± 0.58 for the control group and 1.13 ± 0.21 for the CAD group. Previous studies have reported an MPRi of >2.0 in normal patients. Al-Saadi et al. [13] reported an 87% diagnostic accuracy (sensitivity 90%, specificity 83%) with a MPRi cutoff value of 1.5 for detecting coronary stenosis ≥70%, and Costa et al. [14] showed that a cutoff of 2.04 was 85% sensitive and 49% specific in predicting CAD with ≥50% diameter stenosis. We believe that it is important for each centre to define its own MPRi range, given the impact of many variables, such as contrast dose, stress technique and sequence variations on outcome measures.

Small SDs for between-study differences were detected for perfusion parameters, resulting in small sample size estimates for studies using adenosine stress CMR as a primary endpoint. Syed et al. [9] reported for dobutamine CMR in a cohort of CCS III or IV angina patients, a between-study difference of 1.9 myocardial segments and an SD of 1.6, resulting in a sample size of eight being required to detect a change in two abnormal segments. We show similar favorable findings for adenosine CMR with estimated sample sizes of 12 to detect an absolute change in two ischemic myocardial segments for all subjects, and of 16 for CAD patients.

Commonly used reproducibility measures have potential shortcomings and require cautious interpretation. We used the CoV to define reproducibility, a measure that is expressed as a percentage and not in specific units, thereby allowing comparison of variables using different units or scales. The CoV is calculated from the SD of the differences in measurements divided by the average. Given that, the denominator is dependent on the average value of measurements, the CoV loses utility when the average of measurements approximates zero. This limitation was evident when calculating the CoVs for the number of ischemic myocardial segments in the control group in whom this was, as expected, close to zero. The calculated high CoV of 76% reflects this drawback and should not be interpreted as poor reproducibility. Inter-study variability for the number of ischemic segments in control subjects is in fact low, as shown by the very small mean difference (-0.10) and SD (0.57), as well as the high percentage of perfect agreements (96%) between studies.

### Study limitations

There are several limitations to this study. Currently, CMR does not permit assessment of the entire myocardium. Reproducibility may differ in different myocardial regions and variation in SA slice acquisitions due to variable patient positioning and breath holding can, therefore, affect reproducibility. Furthermore, other groups have performed studies for reproducibility assessment using shorter periods between studies, to minimize physiological variation [11]. Similarly, perfusion CMR was undertaken at two centers and differences in the expertise in these centers may affect data acquisition quality. Reproducibility may have been adversely affected by all of these factors, but nonetheless reflects the practical challenges encountered in both clinical practice and research. Thus, we believe that our assessment of reproducibility by retest at one-to-two week intervals and utilizing two separate imaging centres provides more realistic estimates of the variability to be observed in a longitudinal study. Importantly, our data is limited to a defined perfusion imaging protocol and specific analysis techniques. The use of alternative image acquisition techniques, different contrast agents and dosing, such as dual bolus administration, as well as different techniques for image analysis, such as Fermi deconvolution, may produce significant differences in results for CMR reproducibility. Finally our study was small, based on 10 CAD subjects and 10 controls. This sample size was based on feasibility, and is similar to that used in other typical studies.

## Conclusion

This study demonstrates that adenosine CMR is a reproducible technique with low inter-study and observer variability. Our data provides valuable information for designing adequately powered trials to assess the efficacy of interventions by CMR perfusion and, thus, support the clinical and research applicability of this technique.

## Competing interests

The authors declare that they have no competing interests.

## Authors' contributions

SC and JAM conceived the study. All authors participated in its design. SC and JAM coordinated the study and performed the CMR analysis. SC and ML performed the statistical analysis. SC, JAM, PSM and RMG helped to draft the manuscript. All authors read and approved the final manuscript.
